# The role of immune regulation in HBV infection and hepatocellular carcinogenesis

**DOI:** 10.3389/fimmu.2025.1506526

**Published:** 2025-03-14

**Authors:** Hailong Zheng, Bingchen Xu, Yiyu Fan, Aekkachai Tuekprakhon, Zania Stamataki, Fei Wang

**Affiliations:** ^1^ Department of Hepatobiliary, Pancreatic, and Spleen Surgery, Affiliated Hospital of Inner Mongolia Medical University, Inner Mongolia Medical University, Hohhot, Inner Mongolia Autonomous Region, China; ^2^ Centre for Liver and Gastrointestinal Research, School of Infection, Inflammation & Immunology, College of Medicine and Health, University of Birmingham, Birmingham, United Kingdom

**Keywords:** hepatitis B virus, hepatocellular carcinoma, immune cells, regulatory T cells, HBV infection

## Abstract

Hepatitis B virus (HBV) infection is a well-documented independent risk factor for developing hepatocellular carcinoma (HCC). Consequently, extensive research has focused on elucidating the mechanisms by which HBV induces hepatocarcinogenesis. The majority of studies are dedicated to understanding how HBV DNA integration into the host genome, viral RNA expression, and the resulting protein transcripts affect cellular processes and promote the malignant transformation of hepatocytes. However, considering that most acute HBV infections are curable, immune suppression potentially contributes to the critical challenges in the treatment of chronic infections. Regulatory T cells (Tregs) are crucial in immune tolerance. Understanding the interplay of Tregs within the liver microenvironment following HBV infection could offer novel therapeutic approaches for treating HBV infections and preventing HBV-related HCC. Two viewpoints to targeting Tregs in the liver microenvironment include means of reducing their inhibitory function and decreasing Treg frequency. As these strategies may disrupt the immune balance and lead to autoimmune responses, careful and comprehensive profiling of the patient’s immunological status and genetic factors is required to successfully employ this promising therapeutic approach.

## 1 Introduction

### 1.1 Hepatitis B virus and its global epidemiological characteristics

Hepatitis B virus (HBV) is a pararetrovirus discovered by American geneticist Baruch Blumberg in 1965. HBV is an enveloped, double-stranded DNA virus belonging to the genus *Orthohepadnavirus* and a member of the Hepadnaviridae family. HBV viral particles comprise a double-stranded DNA genome in the form of relaxed circular DNA (rcDNA), containing 3,020–3,320 nucleotides within a capsid made up of hepatitis B core antigen (HBcAg) subunits. This nucleocapsid is encased within a lipid bilayer membrane derived from the host, covered by hepatitis B surface antigen (HBsAg). There are 10 HBV genotypes (A to J), with Genotypes A–D and F being implicated in the development of hepatocellular carcinoma (HCC) (1). The HBV genome contains four overlapping open reading frames (ORFs), namely C, S, P, and X. The C ORF encodes two proteins: the hepatitis B core protein (HBc) and the hepatitis B e antigen (HBe). The S ORF includes the preS1, preS2, and hepatitis B surface antigen (HBs) domains and encodes three envelope proteins, namely the large, middle, and small HBs antigens (HBsAg). The P and X ORFs contain the viral polymerase and HBx genes, which encode the polymerase and viral transcriptional activator HBx proteins, respectively.

HBV infection is the most prevalent chronic viral infection worldwide. The World Health Organization data indicates that 254 million people were living with chronic HBV infection in 2022, with 1.1 million new infections and approximately 1 million deaths, mostly owing to cirrhosis or HCC. HBV was the second leading cause of death from infectious diseases in 2022, second only to COVID-19. In many countries, numerous individuals remain undiagnosed, and even when hepatitis is confirmed, the population that receives treatment is low (2). By the end of 2022, approximately 21% of individuals diagnosed with hepatitis B had received treatment (3). Infection is unevenly distributed worldwide, with sub-Saharan Africa and Southeast Asia being high-prevalence regions. HBV is most commonly transmitted from mother to child at birth (vertical transmission) and from an infected child to a susceptible individual during early childhood (horizontal transmission). The latter carries a 90% risk of developing chronic hepatitis B (CHB) infection (4). The cure for CHB is lacking; however, effective treatments with close monitoring reduce the associated morbidity and mortality (5).

### 1.2 Importance of research in hepatitis B immunology and virology

Hepatitis B infection continues to be a significant global burden, impacting millions of people worldwide. Many individuals with HBV remain undiagnosed, leading to delayed treatment and increased viral transmission. Although an effective vaccine is available, vaccination coverage is thought to be low in certain populations. Current treatments for HBV can manage the infection but do not offer a cure, highlighting the urgent need for the development of curative therapies. It is well known that chronic inflammation plays a crucial role in the onset, promotion, and progression of cancer. Chronic HBV infection are characterized by tissue inflammation with subsequent hepatocellular death, leading to continuous immune activation. Even though immune activation contributes to the restoration of tissue function, a heightened or prolonged immune response may lead to the replacement of hepatic parenchyma by fibrotic tissue and vascular architectural distortion, leading to liver cirrhosis and probably HCC (6, 7). This procedure, first postulated by Virchow back in 1863, is now called the “hepatic inflammation–fibrosis–cancer axis” (8), underscoring the importance of early diagnosis and effective management.

Primary liver cancer is a common malignant tumor worldwide, characterized by insidious onset, long latency period, rapid progression, and poor prognosis, with a 5-year survival rate of only 10–18%, of which HCC accounts for approximately 75–85% (9). Liver cancer ranks sixth in incidence among all malignant tumors worldwide and is the third leading cause of cancer-related mortality (10). CHB infection is a major risk factor for HCC and constitutes approximately 50–80% of HCC cases (11). As HBV infection accounts for most of the global liver cancer deaths; therefore, targeting HBV represents a primary strategy for combatting HCC (12).

HBV infection is broadly categorized into two types, namely acute and chronic hepatitis. HBV infection causes various clinical symptoms, from asymptomatic or mild to severely life-threatening (13). Acute HBV infection is self-limiting and is associated with only a few acute inflammation or hepatocellular necrosis cases and a case fatality rate of 0.5–1% (13, 14). CHBV progresses to advanced liver fibrosis, cirrhosis, and HCC (15), and international guidelines recommend antiviral therapies such as interferon-α (IFN-α) and nucleoside analogs for its treatment (16). Although antiviral therapy has demonstrated a pronounced effect in reducing the incidence of HCC in patients with CHB infection (17–19), the prolonged *in vivo* survival of HBV is caused by the persistent replication of its DNA in hepatocyte nuclei and the resulting low immune system response. In acute HBV infection, the virus is eradicated by the immune system, with the innate and adaptive immune cells playing a crucial role. As HBV infection progresses to a chronic state, it fosters an immune-tolerant microenvironment in the liver by recruiting Foxp3^+^ T regulatory cells (Tregs) and inactivating CD8^+^ T cells, creating favorable conditions for the development of HBV-related HCC (20, 21). Tregs are crucial in maintaining immune tolerance. It is hypothesized that reducing Treg-mediated immunosuppression enhances the quantity and functionality of effector immune cells, improving immune surveillance capabilities (22, 23). Therefore, understanding the interaction mechanisms between the liver microenvironment and immune cells following HBV infection, as well as the regulatory role of Tregs on effector immune cells, may offer new insights for diagnosing and treating HBV infection and HBV-related HCC.

## 2 Hepatitis B viral biology

**Figure 1:**
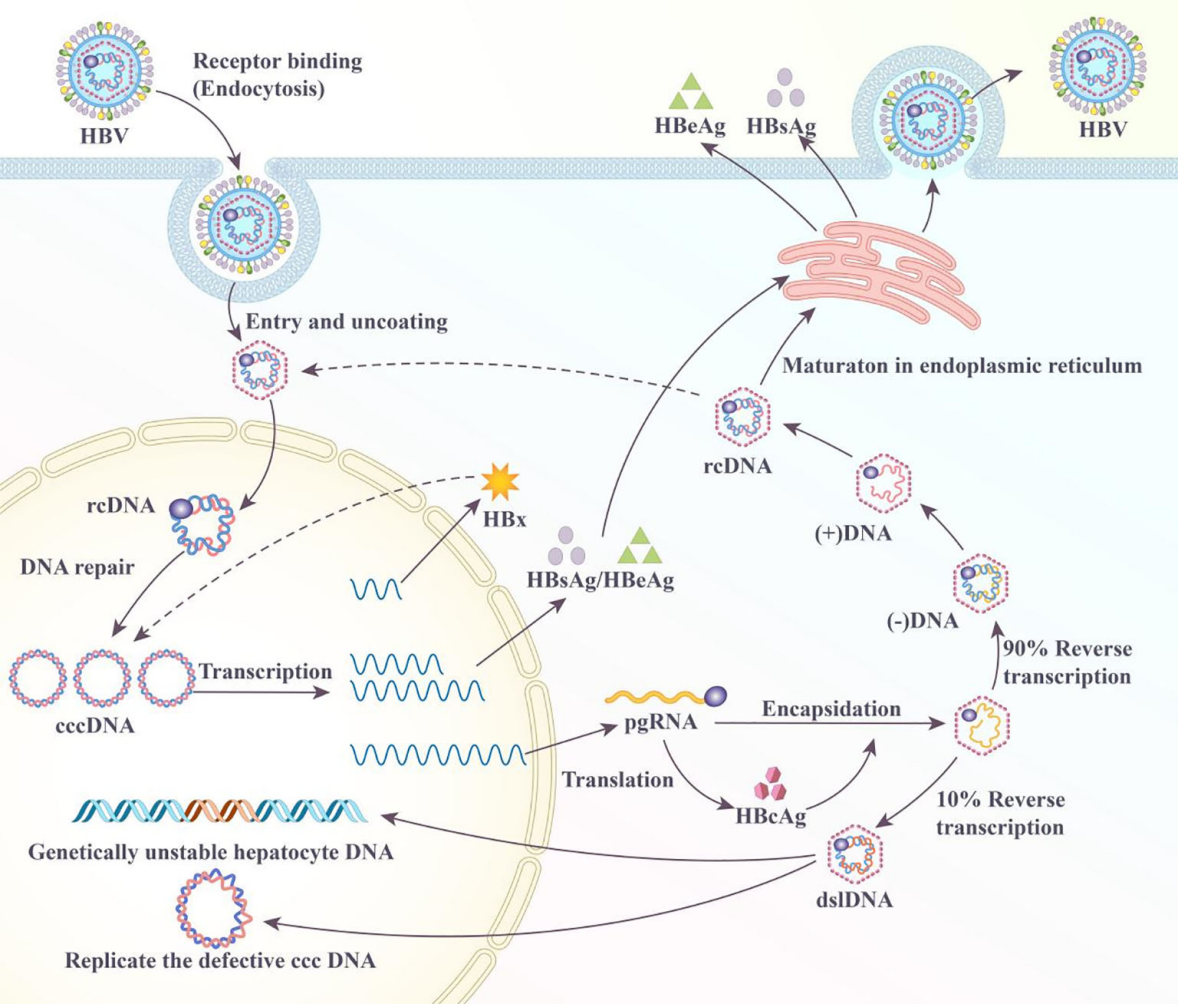
Life cycle of hepatitis B virus (HBV) in the hepatocyte.

HBV is a hepatotropic virus that infects the hepatocytes by binding to the sodium taurocholate cotransporting polypeptide receptor and entering the cell via receptor-mediated endocytosis (24). However, studies have revealed additional hepatocyte receptor proteins, such as transferrin, desialylated glycoprotein, immunoglobulin A receptor, and human hepatic endothelin receptor, as potential binding receptors for HBV (25–29). Upon entering the host cell, HBV releases relaxed circular genomic DNA (rcDNA) into the nucleus. Subsequently, host cell DNA repair enzymes convert HBV rcDNA into the covalently closed circular DNA (cccDNA), which serves as a template for all viral mRNAs (**Figure 1**). The core protein mRNA, known as pregenomic RNA (pgRNA), serves as the template for the core protein and viral DNA polymerase. Once synthesized, the core protein packages the pgRNA into core particles. Within these particles, the viral DNA polymerase reverse-transcribes the pgRNA into rcDNA (**Figure 1**) (30). Core particles containing rcDNA may interact with HBsAg in the intracellular membrane to form mature viral particles that are released from the infected hepatocytes. They may also return rcDNA to the nucleus for repair and the amplification of the cccDNA pool. During the reverse transcription of pgRNA, partial double-stranded rcDNA is formed 90% of the time, with the synthesis of double-stranded linear DNA (dslDNA) constituting 10% of the reverse transcription outcome (**Figure 1**) (31). In contrast to rcDNA, nuclear dslDNA forms replication-deficient cccDNA or integrates into the host cell genome (31–33). Previous studies have shown that integrated HBV DNA may also participate in the synthesis of viral RNA transcripts; however, this integration is not an essential step in the viral life cycle because it does not produce a replication-competent virus (34–36).

HBV dysregulates several pathways in infected hepatocytes, resulting in the development of HCC; however, the exact mechanism is poorly defined. HBV DNA integration reportedly increases genetic instability in infected hepatocytes and is crucial in the progression to HCC (37). A multifunctional HBx protein is significant in HBV pathogenesis and HCC development (38). This HBx protein interferes with multiple signaling pathways, including Wnt/β-catenin and PI3K/Akt/mTOR. The dysregulation of these pathways promotes cell proliferation and survival, resulting in cancerous cell development (38, 39). HBx reportedly interferes with the cell cycle checkpoint, leading to uncontrolled cell division. The HBeAg is among the crucial antigens involved in the HBV infection and the progression to HCC. The presence of HBeAg in the patient’s serum indicates active viral replication with high infectivity, contributing to immune tolerance by inhibiting lymphocyte proliferation and reducing IFN-γ production. HBeAg also impairs T-cell function by increasing programmed cell death-1 (PD-1) and toll-like receptor expression, leading to a weakened immune response and persistent infection. In CHB carriers, HBeAg is associated with liver inflammation and damage, increasing the risk of liver cirrhosis and HCC (40, 41). Given the complex viral biology of HBV and the interplay with host cells, understanding the molecular biology of HBV is crucial in improving the diagnosis, treatment, and prevention of HBV-related diseases, including HCC.

## 3 Roles of innate immune cells in the liver microenvironment following HBV infection

### 3.1 NK cells

**Figure 2:**
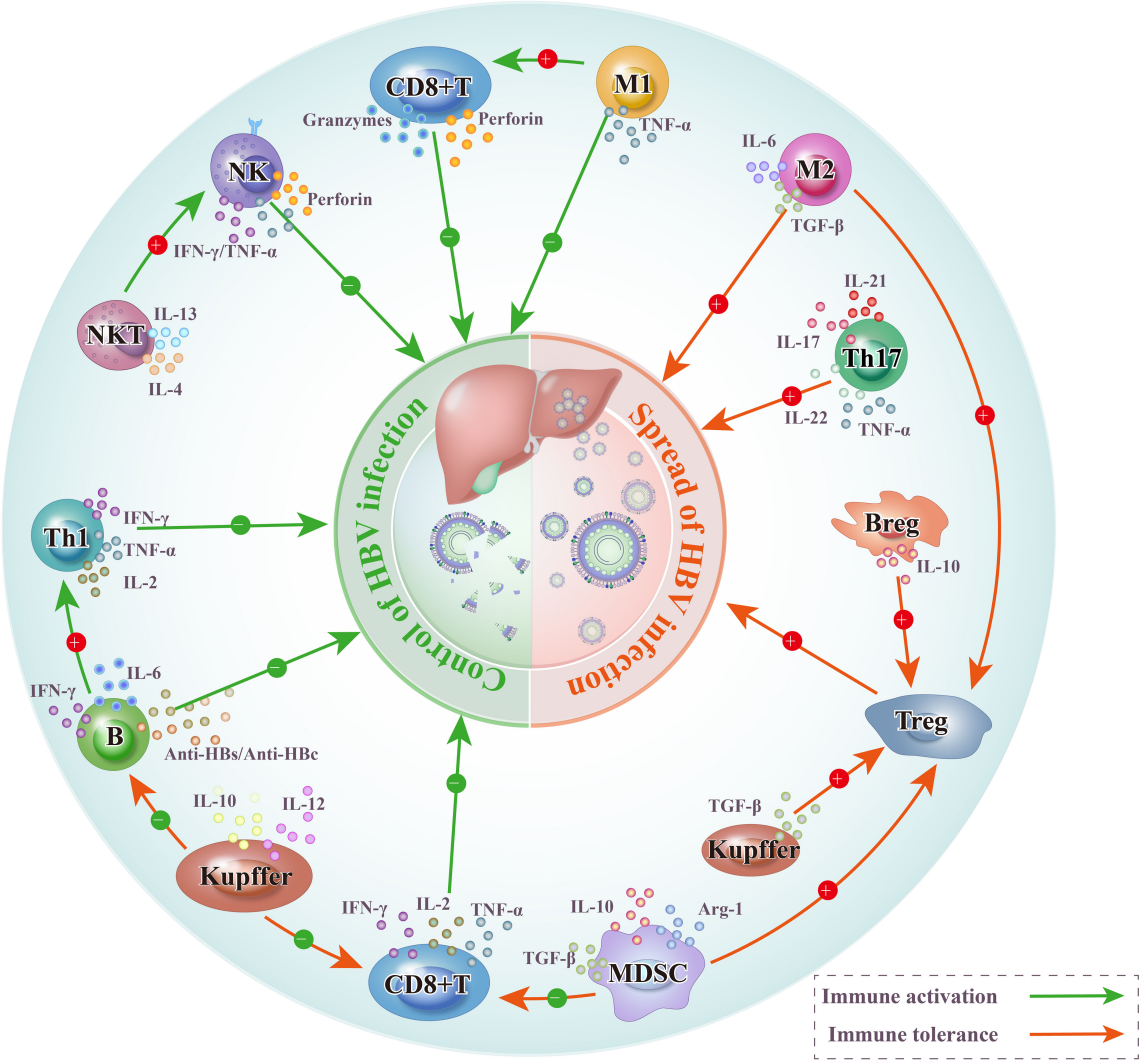
Diagram illustrating how different types of immune cell responses affect the outcome of HBV infection after it enters into liver cells. In the microenvironment of acute HBV infection, the effective response of effector immune cells drives the control of the infection. As HBV infection progresses towards chronicity, the influence of cells that promote immune tolerance leads to a shift from the dominance of effector immune cells to immune-tolerant cells, facilitating the spread of the infection. HBV, Hepatitis B virus; NK, Natural killer cells; NKT, Natural killer T cells; Treg, Regulatory T cells; Breg, Regulatory B cells; B, B lymphocytes; CD8^+^ T, Cytotoxic T lymphocytes; MDSC, Myeloid-derived suppressor cells; Kupffer, Kupffer cells; Th1, Helper T 1 cells; Th17, Helper T 17 cells; M1, M1 macrophages; M2, M2 macrophages.

NK cells constitute 25–50% of hepatic lymphocytes, indicating their central role in innate immunity (42, 43). NK cells are associated with early antiviral and anti-tumor responses (44). In virally infected patients, exposed viral nucleotides, host cell debris, and viral proteins activate various cellular receptors, leading to direct antiviral effects by NK cells through perforin, tumor necrosis factor-related apoptosis-inducing ligand (TRAIL), and the induction of IFN-γ production (**Figure 2**) (45–47). However, HBV may exert immunosuppressive effects by reducing its amplification rate, stimulating TGF-β and IL-10 production and inhibiting the Toll-like receptor 2-induced TNF-α and IL-12 secretion. HBV dissemination within the NK cells is reportedly mediated by HBV-positive exosomes. These exosomes impair NK cell function, affecting IFN-γ production, cytolytic activity, and NK cell proliferation and survival (48). HBV infection induces high IL-10 expression, inhibiting IFN-γ secretion by NK cells and driving the expression of inhibitory receptors PD-1 and CD94 on NK cells. Persistent binding of PD-1/PD-L1 and cytotoxic T-lymphocyte antigen 4 (CTLA4)/CD94 immune checkpoint proteins maintains CD8^+^ T cell stimulation, leading to T cell insufficiency (49). Thus, the NK cell-mediated depletion of antigen-specific CD8^+^ T cells impairs adaptive antiviral immunity in patients with CHB infection and contributes to viral persistence (50). In addition, NK cells retain their cytotoxic potential in CHB infection through the upregulation of TRAIL (51). However, they also target and eliminate HBV-specific CD8^+^ T cells that express high levels of TRAIL death receptors. In CHB infection, NK cells are usually considered more pathogenic than protective, primarily owing to their impaired antiviral cytokine production (52). Therefore, NK cell-based therapeutic strategies should aim to enhance the ability of NK cells to target and destroy virus-infected cells and to reduce the inhibitory effects of NK cells on virus-specific T cells.

### 3.2 Kupffer cells and mononuclear cells

KCs are in the hepatic sinusoids and represent the largest population of hepatic immune cells (53). In the liver microenvironment following HBV infection, KCs and MNCs play a crucial role in creating an immune-tolerant environment that accelerates disease progression (**Figure 2**). KCs facilitate CD8^+^ T cell depletion after HBV infection in mice through interactions with HBcAg (54). A significant mechanism by which CD8^+^ T cells produce IFN-γ is by directing macrophages to produce cytokines and chemokines that promote fibrosis, such as TNF-α, IL-6, and Monocyte Chemoattractant Protein-1 (55). This process contributes to the progression of CHB to HCC. In the liver microenvironment following HBV infection, KCs and MNCs activate the immune response. For example, circulatory CD14^+^ monocytes in patients with CHB infection activate CD8^+^ T cells by upregulating CD137 ligands (55). In mice with CHB infection, the IL-10 released by KCs is crucial for supporting humoral immunity. The dual role of KCs and MNCs on the immune response may be similar to the role of NK cells in CHB infection, both contributing to the protection of hepatocytes from excessive liver inflammation and the maintenance of long-term survival of the organism; however, they affect the antiviral potency. The interplay between KCs and MNCs in the liver microenvironment is critical in shaping the immune response to HBV, usually leading to an immune-tolerant state that benefits virus persistence and disease progression.

### 3.3 Myeloid-derived suppressor cells

MDSCs are a regulatory immune cell population comprising innate immune cells in the liver. A previous study showed that the MDSCs from the liver tissue of HBV transgenic mice suppressed the proliferation of HBsAg-specific allogeneic T cells and lymphocytes (**Figure 2**) (56). The possible cause was the continuous exposure to high HBsAg levels, which induced MDSC expansion, inhibiting the proliferation of effector T cells by promoting the infiltration of Tregs into the liver microenvironment following HBV infection (**Figure 2**) (57, 58). MDSCs express and secrete high TGF-β and IL-10 levels, promoting the peripheral transformation of CD4^+^ T cells to induced Tregs and inducing Treg proliferation (**Figure 2**) (58–61).

## 4 Adaptive immune regulation in the liver microenvironment during HBV infection

Earlier studies have highlighted the crucial role of antigen-specific CD4^+^ and CD8^+^ T cells in preventing viral tropism (62, 63). Effective CD4^+^ helper T cell and CD8^+^ effector T cell responses have been observed in patients with hepatitis (64, 65) and experimentally infected chimpanzees (66) to clear HBV infection. In contrast, B cells exert potent antiviral effects primarily through the production of antiviral antibodies and the presentation of antigens to T cells. With the increased infiltration of Tregs and other immunomodulatory cells in the liver microenvironment following HBV infection, adaptive immune cells, which are crucial for the immune response to HBV, undergo gradual exhaustion (67). As the disease progresses, the resulting liver microenvironment following HBV infection impairs the normal proliferative, transcriptional, epigenetic, and metabolic activities of hepatocytes, significantly increasing the risk of developing HCC (49).

### 4.1 B lymphocytes

As a component of the acquired immune system and a major participant in humoral immunity, B cells are crucial in combating HBV infection. In the early stage of HBV infection, B cells mediate the antiviral response primarily by producing antibodies such as anti-HBs and anti-HBc (**Figure 2**) (68). Additionally, B cells activate T cell-mediated responses by presenting HBcAg to effector T cells (69). Similarly, effector B cells secrete pro-inflammatory cytokines such as IL-6 and IFN-γ, which influence the differentiation of CD4^+^ T cells into Th1 cells (**Figure 2**). As HBV infection progresses to a chronic state, PD-1 expression on specific B cells increases, and these cells exhibit atypical memory phenotypes (CD21^-^ and CD27^-^), resulting in impaired production of anti-HBs antibodies (70). The absence of B-cell antibody production limits HBV clearance, leading to persistent inflammation and promoting the progression of liver disease (71).

The HBV-induced chronic inflammatory microenvironment drives an increased frequency of B cell differentiation into regulatory B cells (Bregs), enhancing the production of immunosuppressive cytokines such as IL-10 and TGF-β (72–74). Bregs orchestrate the immunosuppressive environment to limit tissue damage under chronic inflammatory conditions; however, this allows the immune surveillance system to evade tumors (72–75). Initial studies in the early 2000s attributed this immunomodulation to IL-10, which became the hallmark of Breg suppression (76, 77); however, this notion has lately expanded as new Breg-derived suppressive mediators have been discovered. Human Breg subsets have been identified in various B cell subgroups, and their immunosuppressive functions can be summarized as follows (75): a) IL-10^+^ Bregs inhibit Th1, Th17, and CD8^+^ T cell responses; convert naïve CD4^+^ T cells into regulatory T cell populations; and modulate pro-inflammatory innate cells through the production of IL-10. b) Likewise, TGF-β^+^ Bregs operate on naïve CD4^+^ T cells to generate FoxP3^+^ Tregs, in addition to induce anergy in CD4^+^ and CD8^+^ T cells. c) IL-35^+^ Bregs can promote “infectious tolerance” by inducing IL-35-producing Tregs and expanding the generation of IL-35^+^ Bregs. d) GrB^+^ (Granzyme B) B cells have been shown to inhibit Th1 and Th17 cell responses and to reduce CD4^+^ T cell proliferation by degrading the TCR ζ-chain. Breg cells have been described in various human cancers and are often associated with cancer progression. A higher frequency of IL-10-producing B cells was observed in late-stage esophageal cancer samples compared to early-stage samples, suggesting that Breg cells play a role in the progression of esophageal cancer (78). IL-10 producing B cells were also detected in the tumors of patients with gastric cancer, breast cancer, head and neck squamous carcinoma, and esophageal squamous carcinoma (79–82). B lymphocytes are crucial in combatting HBV infection through antibody production and immune activation; however, the loss of antibody production function in B cells and the increased proportion of Bregs in patients with hepatitis B contribute to HBV-induced liver cancer (**Figure 2**).

### 4.2 CD8^+^ T lymphocytes

During acute HBV infection, HBV-specific CD8^+^ T cells produce pro-inflammatory cytokines such as IFN-γ, IL-2, and TNF-α, as well as cytotoxic molecules, including granzymes and perforins, to control the infection (**Figure 2**) (68). The chronic phase of HBV infection is characterized by various circulating HBV-specific CD8^+^ T cells, including HBsAg-, HBcAg-, and HBV polymerase- CD8^+^ T cells (83). Virus-specific CD8^+^ T cells are crucial in mediating effective antiviral immune responses during CHB infection. However, in CHB infection, HBV-specific CD8^+^ T cells are progressively exhausted. This exhaustion is driven by several mechanisms, including sustained high viral loads, increased Tregs within the liver microenvironment following HBV infection, and the presence of immunosuppressive cytokines such as IL-10 and TGF-β (68, 84, 85). Exhausted T cells are prone to apoptosis owing to the upregulation of TRAIL-2 and the pro-apoptotic mediator Bcl-2 interacting mediator of cell death (50, 86). In addition to diminishing immune surveillance, the exhaustion of CD8^+^ T cells contributes to chronic liver inflammation. During the immune tolerance phase, inflammatory cytokines produced by effector CD8^+^ T cells, such as IFN-γ, TNF, IL-17A, and IL-22, are significantly elevated in patients compared with those in healthy individuals (87). Thus, exhausted CD8^+^ T cells remain partially activated in response to the persistent viral antigen load, inducing ongoing and chronic hepatocellular damage. Chronic liver inflammation accelerates hepatocyte apoptosis, necrosis, and regeneration, resulting in recurrent DNA damage, genomic instability, and mutation accumulation that drive HCC tumorigenesis (88–90). Exhausted CD8^+^ effector T cells impair the adaptive immune system’s surveillance of tumors, allowing tumor cells to evade immune detection (**Figure 2**). Exhausted HBV-specific CD8^+^ T cells characteristically express various co-inhibitory receptors, including PD-1, CTLA-4, T-cell immunoglobulin and mucin domain 3, and T cell immune receptor with Ig and Immunoreceptor Tyrosine-based Inhibitory Motif (ITIM) domains (91–94). The expression of these co-inhibitory receptors in HBV-specific CD8^+^ T cells is associated with high viral loads and reflects the phenotypic and functional characteristics of T cell exhaustion (94). These co-inhibitory receptors, also known as immune checkpoints, have been extensively studied, and some drugs targeting them have shown varying degrees of efficacy in HCC treatment.

### 4.3 CD4^+^ T lymphocytes

Naïve CD4^+^ T cells differentiate into multiple effector T cell subsets. Each subset produces distinct cytokines that drive different immune responses. Th1 cells produce pro-inflammatory cytokines such as IFN-γ, TNF-α, and IL-2, which are vital in anti-tumor and anti-viral immunity by inducing CD8^+^ T cell activation and increasing autoimmune activity (**Figure 2**) (95). HBV-associated HCC tissues exhibit a lower frequency of Th1 cells than do corresponding non-tumor tissues, and a low frequency of Th1 cells is significantly negatively correlated with disease-free survival (96). Th17 cells produce IL-17, IL-21, IL-22, IL-26, and TNF-α. During CHB infection, the balance of circulating helper T cells shifts from Th1 dominance to Th17 dominance (**Figure 2**). As Th17 cells are related to high viral loads and substantial liver injury, this shift towards Th17 dominance is associated with advanced liver disease and a poor prognosis in CHB-related liver disease (97). An increased Th17/Th1 ratio has been identified as an independent predictor of poor disease-free and overall survival in patients undergoing hepatectomy for HCC (96). CD4^+^ cytotoxic T cells secrete granzymes and perforins that exert the direct killing of infected hepatocytes, which depends on the direct recognition of target cells via major histocompatibility complex Class II receptors. In HBV-associated HCC, CD4^+^ cytotoxic T cells are progressively reduced as HCC progresses, and a reduced number and/or functional impairment of CD4^+^ cytotoxic T cells correlates with poor clinical outcomes (98). In patients with HBV-associated HCC, CD4^+^ cytotoxic T cell defects have been linked to a high Treg population (98). Several immunosuppressive mechanisms have been reported for Tregs (99, 100), including the (1) secretion of immunosuppressive cytokines (TGF-β, IL-10, and IL-35), (2) direct killing of effector cells or dendritic cells through granzyme and perforin-mediated cytolysis, (3) suppression of target cells via CTLA4 interactions with CD80/CD86 on T effector cells or dendritic cells, and (4) induction of apoptosis in conventional T cells through profound IL-2 depletion. Thus, Tregs establish an immunosuppressive microenvironment conducive to HBV chronicity and HCC formation by compromising the immunosurveillance functions of the innate and adaptive immune systems.

### 4.4 Regulatory T cells

During viral infection, immune cells release cytokines that are essential for resolving the infection and eliminating infected cells. Tregs are crucial in maintaining immune tolerance and regulating excessive immune activation by preventing immune cells from attacking uninfected self-cells (101–103). Increased Treg frequency inhibits CD8^+^ and CD4^+^ T cell activities, preventing inflammatory liver injury and benefiting the host, and simultaneously shields the virus from immune attack by these T cells (104, 105). This dual effect contributes to disease progression and the development of HCC. An HBV infection study involving mice revealed that in a self-limiting HBV acute infection model, the frequency of liver Tregs briefly peaked before returning to baseline (106). In contrast, in the CHB infection model, Tregs underwent substantial expansion, constituting approximately 20–30% of liver CD4^+^ T cells, and this expansion persisted for over 200 days. HBV-induced hepatic fibrosis showed a similar trend, where the number of Tregs is increased in advanced HBV-associated hepatic fibrosis compared with that in early HBV fibrosis (107). A study (108) involving the measurement of the number of circulating and liver-resident CD4^+^CD25^+^ Tregs in patients with CHB and individuals with HCC showed that the frequency of circulating CD4^+^CD25^+^ Tregs did not differ significantly between them. However, among patients with HBsAg-positive HCC, circulating CD4^+^CD25^+^ Tregs were more frequent than those in individuals with HBsAg-negative HCC, with a 5% difference. Additionally, analysis of Tregs in acute, chronic, and chronic severe HBV infections showed that their numbers were higher in chronic severe HBV infections than in acute or chronic HBV infections (109, 110). These studies demonstrate the significance of Tregs in the pathogenesis of CHB infections and tumor immunity. Increased Tregs in patients with CHB inhibit specific anti-tumor immune responses (**Figure 2**). Furthermore, the HBV transfection of HCC cell lines caused increased frequency of CD4^+^CD25^+^ Tregs and pronounced suppression of tumor antigen-specific CD8^+^ T cell responses. Data from Xus et al. and Franzeses et al. indicated that patients with CHB and higher serum HBV DNA levels had significantly increased circulating CD4^+^CD25^+^ Tregs than did healthy controls, and addressing the exhaustion of these Tregs enhanced antigen-specific CD8^+^ T cell function in those with CHB (109, 111). In the liver microenvironment following HBV Infection, the increased Treg frequency induces immune dysfunction, resulting in viral persistence, chronic tissue damage, cirrhosis, and progression to HCC (**Figure 2**) (109, 112). These results indicate that the frequency of Tregs in the CHB microenvironment is crucial in the progression of viral infection and is significant in preventing the development of CHB-related HCC. Understanding the specific mechanisms by which Tregs inhibit immune cells could be crucial for restoring immune cell activity in the liver microenvironment following HBV infection.

## 5 Tregs and their role in innate and adaptive immunity

### 5.1 Biological functions of Tregs

Tregs include natural Tregs, which emerge from the thymus, and induced from the peripheral inflammatory microenvironment (113). The induced Tregs are the predominant types in tumors (114) and are classified into three subpopulations, namely Tr1, Th3, and iTR35. Tr1 subpopulation: This subset is characterized by CD4, CD18, CD49b, LAG3, and GATA3 expression, while lacking FOXP3 and exhibiting relatively high CD25 levels (115). Tr1 cells are functionally impaired *in vitro*; they suppress immune cell proliferation by producing IL-10 (116). Th3 subpopulation: they produce TGF-β, IL-4, and IL-10. A study suggests (117) that Th3 cells may also express the surface molecules typically on natural Tregs, such as CD25, FOXP3, and CTLA4. The iTR35 subpopulation is IL-35-producing CD4^+^ T cells. IL-35, which comprises two subunits, IL-12p35 and Ebi3, reportedly promotes the development of Tregs that mediate suppression in an IL-35-dependent manner (118). Under homeostatic conditions, Tregs exhibit reduced recirculation and tissue infiltration compared with those of conventional T cells. However, under pathological conditions, Tregs show increased recirculation to inflamed, tumor, and infection sites through lymphoid tissues (119). In humans and other mammals, Tregs prevent autoantigenic responses by suppressing innate (e.g., NK cells) and adaptive (e.g., T and B lymphocytes) immune cells (120, 121). Releasing the Treg-induced suppression of immune cells enhances the immune response of these cells against HBV or tumors (111, 122). Therefore, understanding the specific mechanisms of Treg interactions with immune cells could be beneficial for controlling HBV infection and treating HCC.

### 5.2 Role of Tregs in the regulation of effector T and B cells

**Figure 3:**
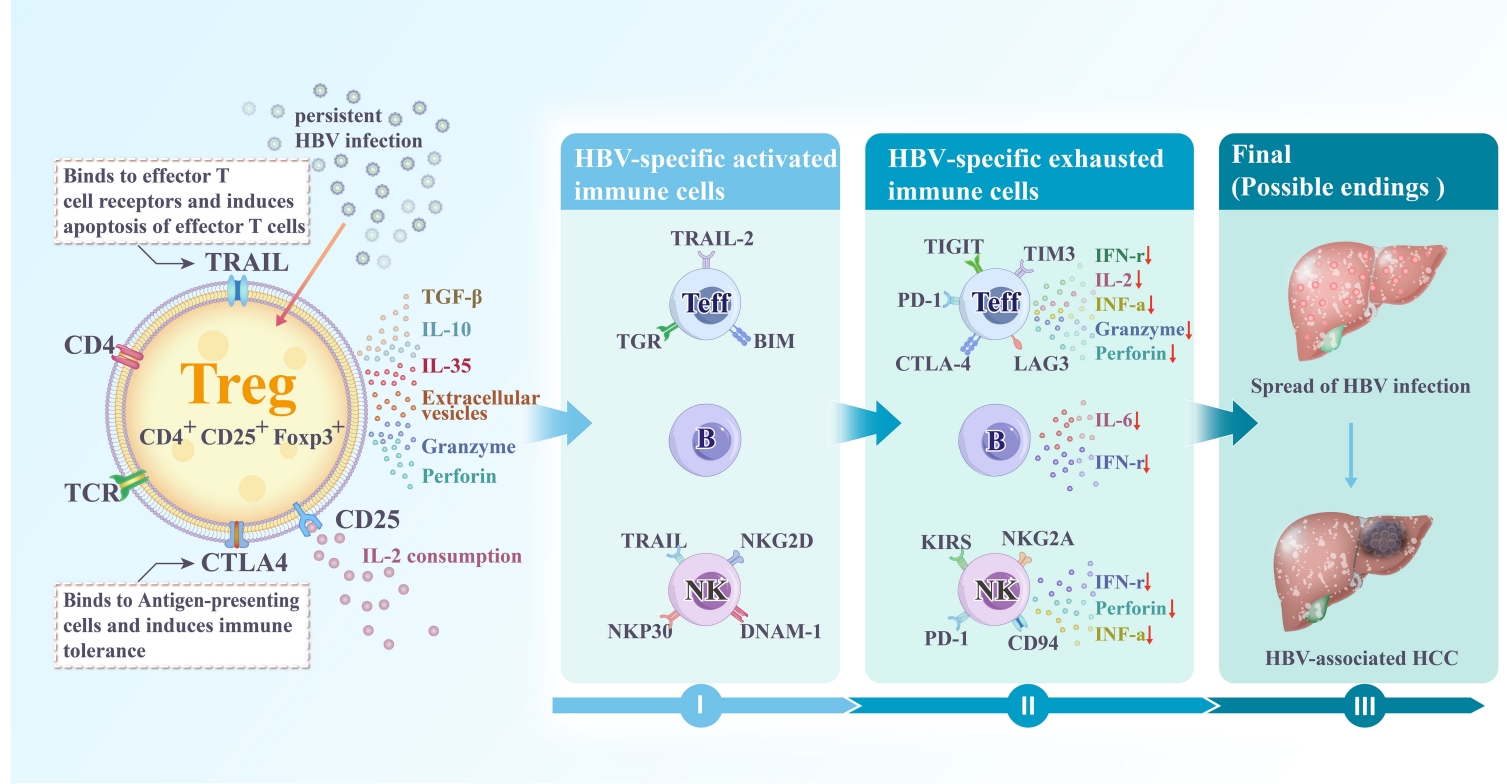
Chronic HBV infection leads to the differentiation of CD4^+^ CD25^+^ T cells into CD4^+^ CD25^+^ FOXP3^+^ regulatory T cells (Tregs). Although the function of Tregs is crucial for limiting host damage, persistent HBV infection causes Tregs to promote the conversion of effector immune cells to an immune-tolerant phenotype, creating an immunosuppressive microenvironment prone to tumors. This occurs through: (1) the production of suppressive cytokines; (2) direct killing of effector cells via perforin and granzyme; (3) direct inhibition of antigen-presenting cells through CTLA4-CD80/86 interactions; (4) consumption of IL-2 via CD25; (5) secretion of extracellular vesicles that inhibit effector immune cells; (6) direct killing of effector cells by TRAIL binding to death receptors DR4 (TRAIL-RI) and DR5 (TRAIL-RII) on their surface. HBV, Hepatitis B virus; Treg, Regulatory T cells; Teff, Effector T cells; B, B lymphocytes; NK, Natural killer cells; HCC, Hepatocellular carcinoma; IL, Interleukin; TGF, Transforming growth factor; IFN, Interferon.

It is well established that the number and function of anti-infective and anti-tumor immune cells are determinants of disease progression, among which effector T cells are crucial. Tregs primarily function is to maintain immune tolerance and prevent peripheral autoimmunity through the secretion of immunosuppressive cytokines (TGF-β, IL-10, and IL-35), the expression of CTLA4, and competition for IL-2, which inhibits the secretion and proliferation of cytokines (granzymes A and B, perforin, and γ-interferon) from effector T cells to maintain immune tolerance and prevent autoimmunity in the periphery (**Figure 3**) (123). Studies have also shown that Tregs directly kill effector immune cells through the galectin-1-, granzyme B-, and TRAIL pathways, inhibiting their anti-infection and anti-tumor activities (99, 100). Recent studies revealed that Tregs secrete extracellular vesicles (EVs) as a novel inhibitory mechanism, capable of modulating the immune response in a manner independent of cell contact or targeting. Treg-derived EVs suppress effector T cell-mediated responses by transferring packaged Let-7d miRNA to Th1 cells, inhibiting their proliferation and IFN-γ secretion (124). Aiello et al. further demonstrated that EVs derived from Tregs convert T cells into Tregs by delivering miR-503, miR-330, and miR-9 (125). Exposure to these Treg-derived EVs results in an increased secretion of IL-10 and the expression of T lymphocyte immunoglobulin mucin 3 in naïve T cells, indicating that these EVs regulate T cell behavior. Treg-derived EVs effectively inhibited T cell proliferation in a dose-dependent manner (126). In addition to their effects on effector T cells, Tregs demonstrate their relationship with adaptive immunity by inhibiting the antigen presentation of B cells to self-reactive T cells. For example, Tregs inhibit the secretion of pro-inflammatory cytokines such as IL-6 and IFN-γ by effector B cells to regulate the differentiation of CD4^+^ T cells to Th1 and inhibit the activation of cytotoxic T cells through the reduction of IL-2, suppressing immune function (**Figure 3**) (71).

### 5.3 Role of Tregs in the regulation of NK cells

In the early stages of HBV infection, NK cells play a primary role in immune defense. However, as the disease progresses to a chronic state and transforms into HCC, NK cells exhibit reduced cell frequency and decreased IFN-γ production, reflecting diminished cytotoxic activity (**Figure 3**) (127, 128). Enhancing the cytotoxic activity of NK cells improves the overall and relapse-free survival in HCC (129). The above studies suggest that NK cells play a central role in innate immunity. For example, monalizumab, a humanized antibody targeting the inhibitory receptor NKG2A on NK cells, reportedly enhances NK cell activity against tumor cells. This effect is further amplified when combined with immune checkpoint blockade (PD-1/PD-L1), which helps restore CD8^+^ T cell function. Similarly, the role of Tregs in suppressing NK cell function has been shown in animal models (130). In this model, reducing Treg frequency by administering an anti-CD25 monoclonal antibody before tumor inoculation limited tumor growth and promoted cytotoxic cell production, predominantly NK cells (131). Tregs reduced IL-2 levels in the liver cancer microenvironment through competitive uptake; however, IL-2 enhanced NK cell function and decreased lung metastasis of liver cancer in mice (132, 133). The inhibition of Tregs led to reduced TGF-β and IL-10 expression, enhancing NK cell function and increasing anti-infective and anti-tumor activities (130). Additionally, Tregs produce granzyme B and perforin, which induce NK cell apoptosis and contribute to disease progression (134). Their relationship has also been validated in other common liver diseases beyond HCC. NK cells and Tregs increase in parallel during acute episodes of liver inflammation and autoimmune liver disease (135), suggesting that inhibiting Tregs could enhance NK cell function.

## 6 Current research progress and prospects on HBV therapy

The primary treatments for HBV are IFN-α and nucleoside analogs. However, these therapies have significant limitations, including their inability to completely eradicate HBV, implying that patients usually require lifelong treatment to manage infection. Additionally, the functional cure (HBV DNA suppression and loss of HBsAg levels with or without the detection of anti-HBsAg antibody) is rarely achieved after treatment with nucleoside/nucleotide analogs; however, this occurs in approximately 10% of patients who receive pegylated interferon therapy (136). Failure of the functional cure usually leads to CHB-associated HCC, particularly in patients with cirrhosis. This underscores the need for more effective therapies to prevent cancer progression. This is among the pivotal approaches for improving patient outcomes.

### 6.1 Effector T cell-based HBV immunotherapy

In patients with HBV-associated HCC, chimeric antigen receptor T-cells (CAR-T) and T-cell receptor-engineered T-cells targeting HBV antigens have shown antiviral and anti-HCC activities *in vitro* (137, 138). A study (139) of CAR-T therapy showed that five out of six patients with CHB-associated HCC lung metastases treated with HBV-specific CAR-T cells for 1 year experienced a reduction in lung metastases. Additionally, these cells did not adversely affect liver function over the same period. Mucosal-associated invariant T (MAIT) cells, which are naturally abundant in the liver, represent a significant subset of innate-like T cells with potent effects within the hepatic environment (140, 141). Healy et al. explored the redirection of T-cell receptors to MAIT cells in the HBV using a preclinical HCC cell model. Their findings support the potential use of MAIT cells in liver-targeted immunotherapy for HBV-associated HCC (142).

### 6.2 NK cell-based HBV IMMUNOTHERAPY

NK cell exhaustion leads to decreased cytotoxicity and impaired cytokine production (143). NK cells are significant components of the human innate immune system. The absolute numbers of circulating and intrahepatic NK cells are positively correlated with the control of HBV infection and the survival and prognosis of patients with HBV-associated HCC (144, 145). To enhance the targeting and effectiveness of NK cells against HBV and tumor cells, various genetic engineering strategies have been developed. The techniques used to create CAR-T cells have also been adapted for NK cells, improving the specificity and efficacy of NK cell therapy (146, 147).

### 6.3 Treg-based immunotherapy

Tregs are among the most prevalent suppressor cells in the HBV-associated tumor microenvironment. Tregs play a crucial role in suppressing effector immune cells and influencing disease prognosis in patients with hepatitis B by maintaining the immune-tolerant microenvironment established by HBV infection (109, 111, 148). There are two approaches to targeting Tregs in the liver microenvironment following HBV Infection, namely reducing their inhibitory function and decreasing Treg frequency.

#### 6.3.1 Reducing Treg inhibitory functions

The evident breakthrough in immunotherapy has been the use of immune checkpoint inhibitors (ICIs), antibodies that block key immunomodulatory molecules such as CTLA-4, as well as PD-1 and its ligand PD-L1. Notably, Tregs express many checkpoint molecules, including CTLA-4, PD-1, OX40, and LAG-3, making them direct targets for ICI immunotherapy (149). A recent meta-analysis (150) demonstrated that patients with advanced HCC treated with ICIs had a low risk of HBV infection and that ICI therapy can be safely administered to individuals with concurrent HCC and CHB. This study highlights the feasibility and significant therapeutic potential of ICI immunotherapy against HBV infection.

The stable expression of FOXP3 is associated with the demethylation of a regulatory region known as the Treg-specific demethylated region (FOXP3-TSDR), which helps to maintain the suppressive phenotype and ensures the expression of the Treg-specific gene (151). Using tools such as CRISPR/Cas9 to precisely modify the FOXP3-TSDR in Tregs enhances their suppressive function in autoimmune diseases. This could also be used as the target to reduce their immunosuppressive effects in cancer therapy (152). In addition, FOXP3 mRNA was proven to be targeted by miR-1231, miR-31, and miR-647. Targeting FOXP3 mRNA may effectively reduce the suppressive effects of Tregs, enhancing the immune function (153). Recent data showed that targeting the CD27/CD70 co-signalling axis affects Treg functions. CD27+CD70+ Tregs specifically show low levels of DNA methylation in TSDR, indicating the suppressive function of Tregs; however, CD27−CD70+ Tregs exhibit high DNA methylation levels in the TSDR region, which affects their function. Tregs with high CD27 expression tended to maintain high levels of FOXP3 expression, whereas CD70 expression was negatively correlated with FOXP3 expression levels (154). Thus, understanding and manipulating the CD27/CD70 interaction could provide novel approaches to controlling immune responses, which could be beneficial in treating various immune-related conditions.

#### 6.3.2 Reducing the frequency of Tregs

In 2019, Zania et al. discovered a unique intracellular structure termed Enclysis, which refers to hepatocyte-specific phagocytosis of active CD4^+^ T cells (155). This structure regulates the number of Tregs. Enhancing Enclysis reduces Treg frequency, alleviates local immunosuppression in the liver microenvironment following HBV Infection, and restores immune cell activity. This process occurs in normal hepatocytes, hepatocellular carcinoma cells, and isolated liver samples. Therefore, understanding how Enclysis regulates the frequency of Treg cells in the liver is crucial for the treatment of chronic liver inflammation and HBV-associated HCC.

Hepatocytes selectively engulf CD4^+^ T cells rather than CD8^+^ T or B cells. This selectivity may be because CD8^+^ T and B cells migrate through hepatocytes paracellularly, while Tregs enter hepatocytes via vesicles, where they are acidified and degraded. The interaction between CD4^+^ T cells and hepatocytes can be divided into three primary stages, namely early adhesion, firm binding, and spontaneous migration. The binding of CD4^+^ T cells to hepatocytes relies on intercellular adhesion molecule-1, which facilitates early adhesion and the subsequent internalization of T cells. β-Catenin, a key molecule in cellular structure for epithelial cells, also plays a crucial role in the formation of Enclysis vesicles. Similarly, the formation of Enclysis vesicles shares similarities with endocytosis, as both processes rely on the actin cytoskeleton to transport vesicles from the extracellular space into the cell. The key difference is that endocytosis is a non-specific process, whereas hepatocytes exhibit a high degree of specificity for CD4^+^ T cells (155, 156). Therefore, Enclysis is a natural process that potentially alters Treg frequency in the liver. This is crucial for immune regulation in the liver and introduces a novel, specific mechanism for modulating Treg frequency in the liver microenvironment following HBV Infection. Although the molecular mechanisms underlying the regulation of Enclysis in the liver are partially understood, the discovery of this phenomenon may be significant for the future of immunotherapy in liver diseases.

The advent of immunotherapy has improved outcomes in patients with HCC; however, its application in HBV remains limited. Immunotherapy aims to boost the number and function of immune effector cells, such as effector T and NK cells, while inhibiting immunosuppressive cells, such as Tregs, within the liver microenvironment following HBV infection. A primary challenge is translating these promising strategies from research into clinical practice, which involves extensive clinical trials to ensure safety and efficacy. Personalized approaches to tailor the treatments based on a patient’s specific tumor profile and immune status, as well as genetic factors, are essential and pose the key challenge for treatment development. This requires the combination of advanced diagnostic tools and biomarkers to identify the most effective treatment. Similarly, immune evasion is a major challenge in immunotherapy, as HBV infection and cancer cells downregulate antigen presentation, activate immune checkpoints, and create an immunosuppressive tumor microenvironment.

## 7 Summary

The interplay between immune tolerance and effector immune cells is essential for maintaining the liver's immune microenvironment, with their regulation being crucial for sustaining immune homeostasis. In the context of HBV infection and related diseases, inhibiting immune tolerance cells or enhancing the quantity and function of effector immune cells is a significant focus in immunotherapy research. Tregs, as key mediators of the immune-tolerant microenvironment in HBV-infected livers, are intricately linked to other hepatic immune cells. The proposed approach emphasizes reducing Treg frequency while simultaneously enhancing the functionality of effector immune cells to support a comprehensive immune response. This strategy could bolster both innate and adaptive immunity against HBV infection and suppress HBV-related HCC. However, removing or depleting Tregs in the liver presents significant challenges, including risks of severe inflammation, autoimmunity, and immune overactivation. Precise targeting is essential to avoid systemic effects and compensatory mechanisms that might diminish therapy effectiveness. Balancing safety and efficacy, understanding long-term consequences, and managing potential side effects are crucial for developing effective Treg-targeted therapies in the liver.
